# Strong localization of oxidized Co^3+^ state in cobalt-hexacyanoferrate

**DOI:** 10.1038/s41598-017-16808-1

**Published:** 2017-11-29

**Authors:** Hideharu Niwa, Masamitsu Takachi, Jun Okamoto, Wen-Bin Wu, Yen-Yi Chu, Amol Singh, Di-Jing Huang, Yutaka Moritomo

**Affiliations:** 1 Faculty of Pure and Applied Science, University of Tsukuba, Tsukuba, 305-8571 Japan; 2 Graduate School of Pure and Applied Science, University of Tsukuba, Tsukuba, 305-8571 Japan; 3 National Synchrotron Radiation Research Center, Hsinchu, 30076 Taiwan; 4 Tsukuba Research Center for Energy Materials Science (TREMS), University of Tsukuba, Tsukuba, 305-8571 Japan

## Abstract

Secondary batteries are important energy storage devices for a mobile equipment, an electric car, and a large-scale energy storage. Nevertheless, variation of the local electronic state of the battery materials in the charge (or oxidization) process are still unclear. Here, we investigated the local electronic state of cobalt-hexacyanoferrate (Na_*x*_Co[Fe(CN)_6_]_0.9_), by means of resonant inelastic X-ray scattering (RIXS) with high energy resolution (~100 meV). The *L*-edge RIXS is one of the most powerful spectroscopic technique with element- and valence-selectivity. We found that the local electronic state around Co^2+^ in the partially-charged Na_1.1_Co^2+^_0.5_Co^3+^_0.5_[Fe^2+^(CN)_6_]_0.9_ film (*x* = 1.1) is the same as that of the discharged Na_1.6_Co^2+^[Fe^2+^(CN)_6_]_0.9_ film (*x* = 1.6) within the energy resolution, indicating that the local electronic state around Co^2+^ is invariant against the partial oxidization. In addition, the local electronic state around the oxidized Co^3+^ is essentially the same as that of the fully-charged film Co^3+^[Fe^2+^(CN)_6_]_0.3_[Fe^3+^(CN)_6_]_0.6_ (*x* = 0.0) film. Such a strong localization of the oxidized Co^3+^ state is advantageous for the reversibility of the redox process, since the localization reduces extra reaction within the materials and resultant deterioration.

## Introduction

Lithium-ion/sodium-ion secondary batteries (LIBs/SIBs) are important energy storage devices for a mobile equipment, an electric car, and a large-scale energy storage. The device stores electric energy as material energy through a reversible redox process in cathode and anode materials. To comprehend what happens in battery materials in the charge (oxidization) process, we should know variation of the local electronic state in a valence-selective manner. In other words, we should clarify how far the effect of the oxidized site spreads and what kind of electronic state the oxidized site is.

**Figure 1:**
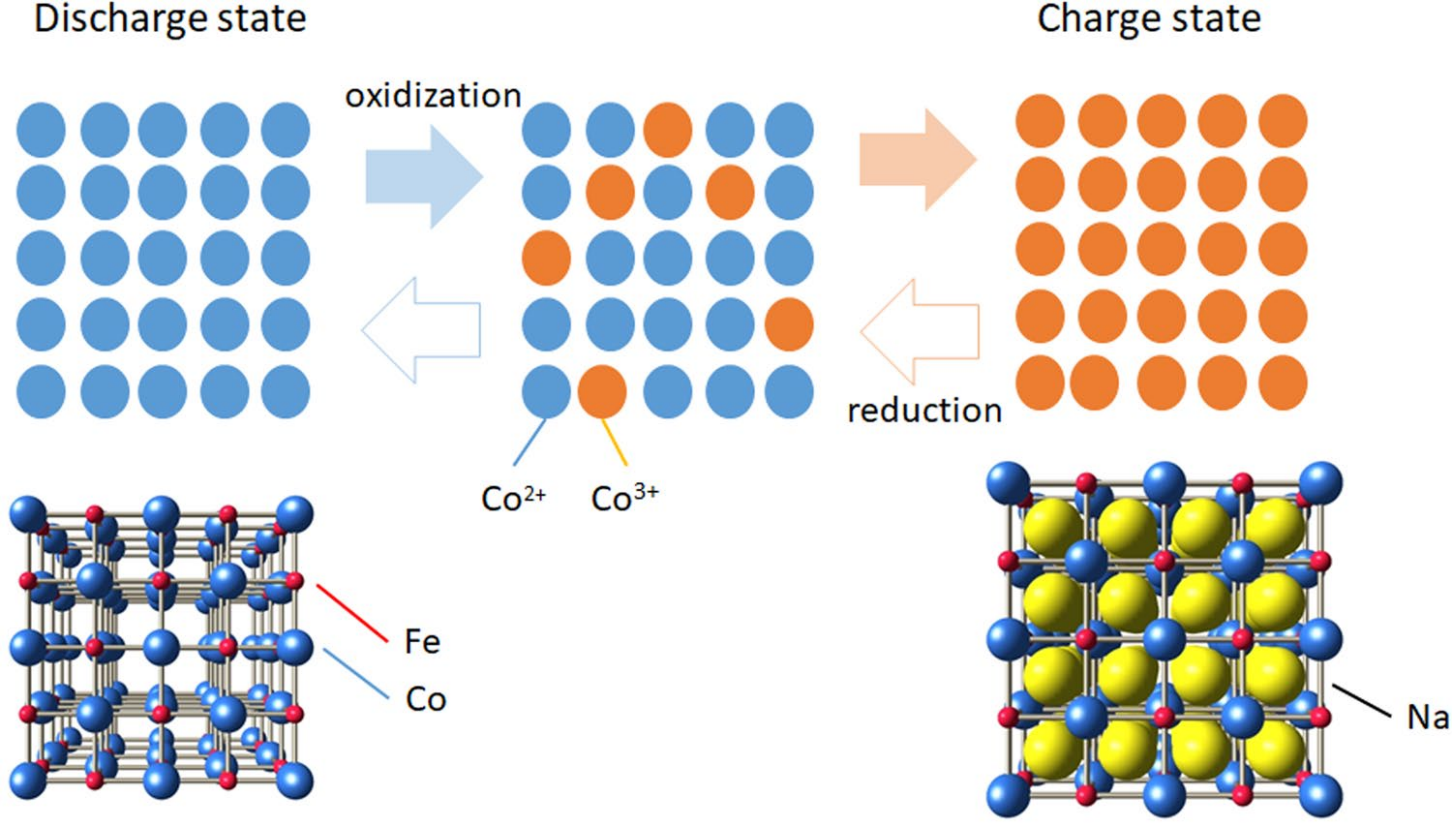
Schematic illustration of redox process in cobalt-hexacyanoferrate. The bottom illustrations show schematic crystal structure in the charged and discharged states. Bars represent cyano groups (CN).

Among the cathode materials, the metal (*M*) - hexacyanoferrates (Na_*x*_ *M*[Fe(CN)_6_]_y_^1^) are attracting current interest of material scientists, because they are promising cathode materials for LIBs^2–4^ and SIBs^5–15^. The *M*-hexacyanoferrates consist of three-dimensional jungle-gym type -*M*-NC-Fe-CN-*M*-NC- Fe-CN-*M*-NC- network and Na^+^ and H_2_O, which are accommodated in the network nanopores. Most of the *M*-HCFs show the face-centered cubic (*Fm* $\overline{3}$ *m*; *Z* = 4). Figure 1 shows schematic illustration of the redox process in cobalt-hexacyanoferrate. The constituent Co ions take the divalent high-spin (HS) state in the discharge state. In the charge (oxidization) process, the Co sites are selectively oxidized with deintercalation of Na^+^. Eventually, all the Co sites are oxidized in the charge state, where the Co sites take trivalent low-spin (LS) state^5^. The charge state is unstable, and hence, the discharge (reduction) process spontaneously takes place when the battery are connected to an external load.

In the actual compounds, the charge process accompanies significant structural change, *e.g*., volume expansion/shrinkage. For example, the lattice constant (*a*) of Na_*x*_Co[Fe(CN)_6_]_0.9_ steeply decreases with charge process from 10.2 Å at *x* = 1.6 to 9.9 Å at *x* = 0.8, because the ionic radius (*r*_HS_ = 0.745 Å) of HS Co^2+^ is much larger than that (*r*_LS_ = 0.545 Å) of LS Co^3+^ (Fig. 1). Such a severe structural change is considered to influence the local electronic states. We note that Na_*x*_Co[Fe(CN)_6_]_0.9_ has considerable Fe(CN)_6_ vacancies, where H_2_O molecules coordinate to Co instead of CN. Takachi *et al*.^5^ systematically investigated the structural and electronic properties of Na_*x*_Co[Fe(CN)_6_]_0.9_ against Na^+^ concentration (*x*). The crystal structure remains face-centered cubic in the whole region of *x* (0.0 < *x* < 1.6) without showing phase separation nor phase transition. This suggests that the oxidized Co^3+^ sites are uniformly distributed. The X-ray absorption spectroscopy (XAS) around the Co K-edge suggests coexistence of HS Co^2+^ and LS Co^3+^ in the region of 0.6 < *x* < 1.6. Further analyses of the XAS, however, are impossible due to lack of valence-selectivity. The *L*-edge resonant inelastic X-ray scattering (RIXS) with high energy resolution (~100 meV) enable us to detect even a slight variation of the local electronic state around the Co site in a valence-selective manner.

Here, we investigated how the local electronic state around the Co cite changes in the charge process in Na_*x*_Co[Fe(CN)_6_]_0.9_ by means of the Co *L*_3_-edge RIXS with high energy resolution. The RIXS revealed that the local electronic state around Co^2+^ is invariant within the energy resolution against the partial oxidization. In addition, the local electronic state around the oxidized Co^3+^ is essentially the same as that of the fully-charged film (*x* = 0.0). The localization of the oxidized Co^3+^ state, which is probably stabilized by the heterogeneous lattice structure, is advantageous for the reversibility of the redox process.

## X-ray absorption spectra around the Co L_3_-edge

We prepared three Na_*x*_Co[Fe(CN)_6_]_0.9_ films with different Na^+^ concentration (*x*) by means of the electrochemical method. In Table 1, we listed the *x*-controlled Na_*x*_Co[Fe(CN)_6_]_0.9_ films together with the nominal valence state of Co and Fe. For convenience of explanation, we will call the films as the HS Co^2+^ (*x* = 1.6), mixed (*x* = 1.1), and LS Co^3+^ (*x* = 0.0) films, respectively.

**Table 1:** List of the *x*-controlled Na_*x*_Co[Fe(CN)_6_]_0.9_ films with nominal valence state.

name	*x*	nominal valence state	chare/discharge state
HS Co2^+^	1.6	Na_1.6_Co^2+^[Fe^2+^(CN)_6_]_0.9_	Discharged
mixed	1.1	Na_1.1_Co^2+^_0.5_Co^3+^_0.5_[Fe^2+^(CN)_6_]_0.9_	partially-charged
LS Co^3+^	0.0	Co^3+^[Fe^2+^(CN)_6_]_0.3_[Fe^3+^(CN)_6_]_0.6_	Charged

**Figure 2:**
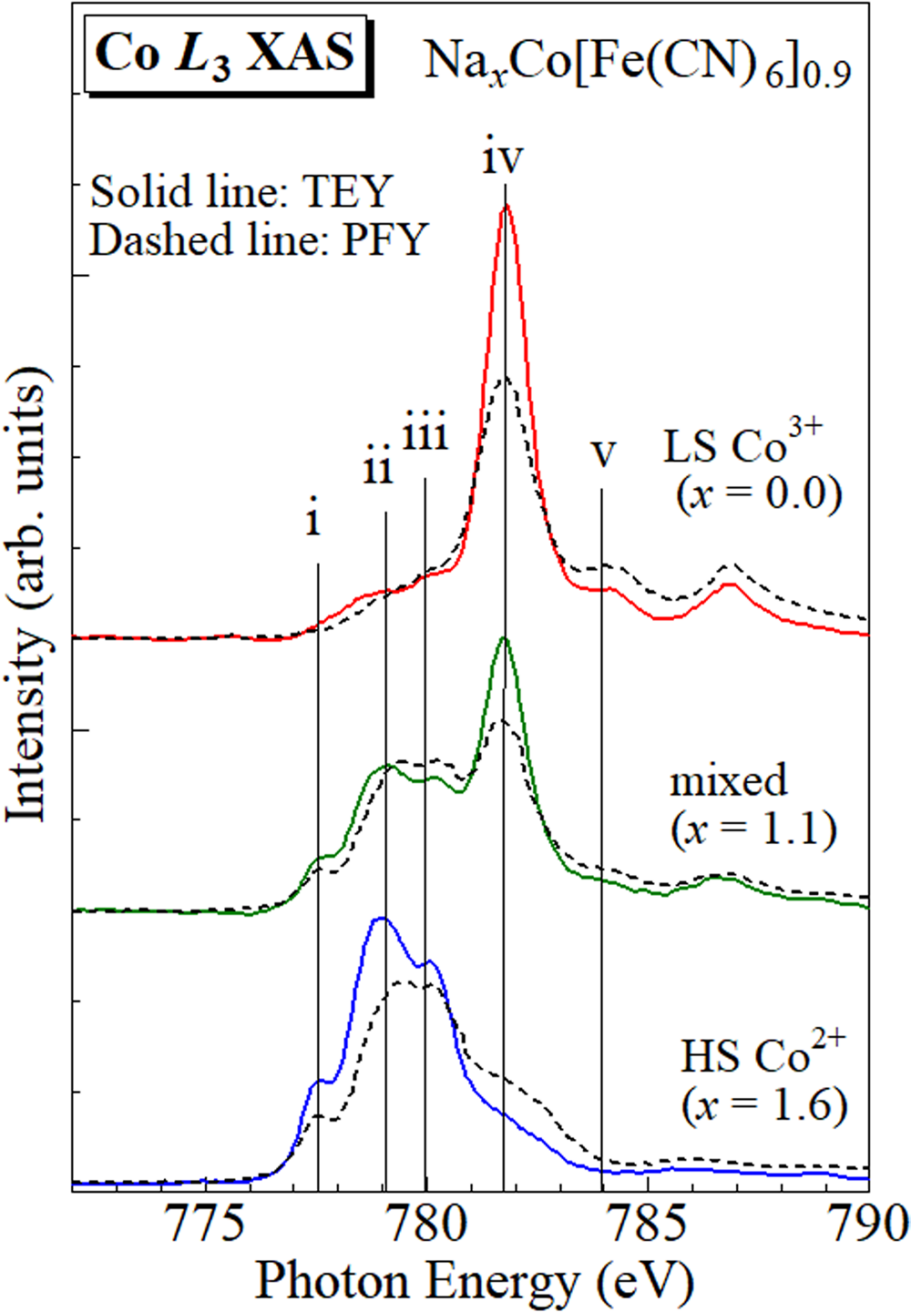
X-ray absorption spectra around the Co *L*_3_-edge of the Na_*x*_Co[Fe(CN)_6_]_0.9_ films against *x*. Solid and dashed lines represent the TEY and PFY spectra, respectively. Vertical lines (i–v) represent the incident photon energies in the RIXS measurements (*vide infra*).

Figure 2 shows absorption spectra around the Co *L*_3_-edge of the three films. The measurements were performed at Taiwan Light Source (TLS) BL08B1 beamline at the National Synchrotron Radiation Research Center (NSRRC) in Taiwan. The spectra indicated by solid and dashed curves were obtained in the total electron yield (TEY) and partial fluorescence yield (PFY) modes, respectively. The TEY mode is surface-sensitive. In all the films, the peak features of the TEY spectra are similar to the PFY spectra.

In the HS Co^2+^ film (*x* = 1.6), the TEY spectrum shows characteristic peaks around 777.5, 779, and 780 eV. The spectral feature is similar to that of CoO which serves as HS Co^2+^ ref.^16^. In the LS Co^3+^ film (*x* = 0.0), the TEY spectrum consists of sharp peak at higher energy around 782 eV with a shoulder structure around 784 eV. The spectral feature is similar to that of EuCoO_3_ which serves as LS Co^3+^ ref.^16^. The TEY spectrum of the mixed film (*x* = 1.1) is close to the superimposed spectrum of those of the HS Co^2+^ and LS Co^3+^ films. This suggests coexistence of the HS Co^2+^ and LS Co^3+^ sites in the mixed film. In Fig. S2, we show the absorption spectra around the Co *L*_2,3_-edge of the three films.

## RIXS spectra around the Co *L*_3_-edge

**Figure 3:**
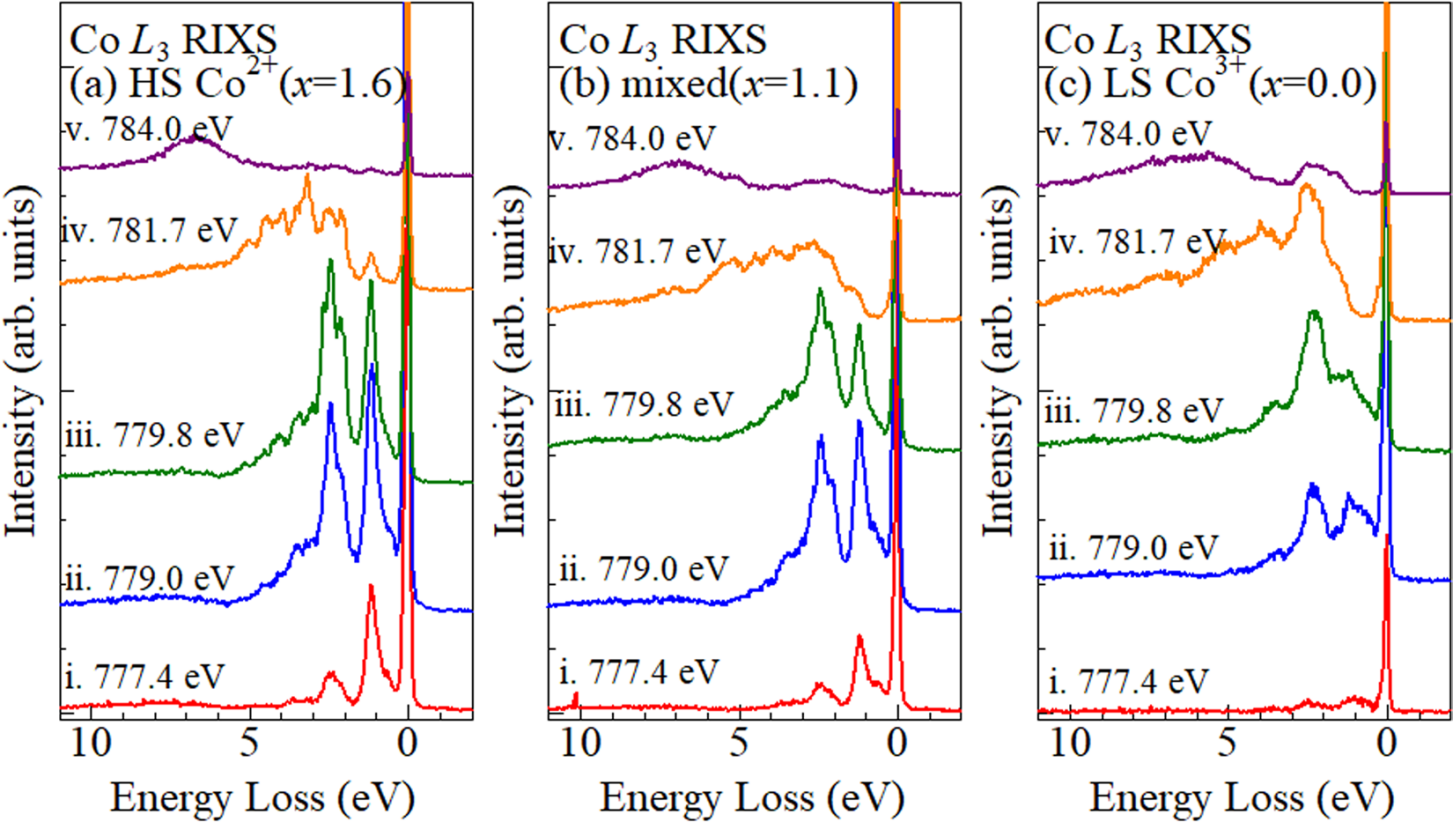
RIXS spectra around the Co *L*_3_-edge of the Na_*x*_Co[Fe(CN)_6_] films: (**a**) *x* = 1.6, (**b**) 1.1, and (**c**) 0.0. The spectra were normalized to the incident photon flux.

Figure 3 shows the RIXS spectra around the Co *L*_3_-edge of the three films: (a) HS Co^2+^ (*x* = 1.6), (b) mixed (*x* = 1.1), and (c) LS Co^3+^ (*x* = 0.0). The measurements were performed at TLS BL05A1 beamline^17^ at the NSRRC in Taiwan. The horizontal axis represents the energy loss of the scattered X-ray, which are mainly transferred to the crystal-field excitations of Co. The excitation photon energies (*E*_ex_) are indicated by vertical lines (i–v) in Fig. 2. The spectra were normalized to the incident photon flux. As discussed above, the RIXS spectra at *E*_ex_ < 779.8 eV are dominated by the scattering due to HS Co^2+^ while the spectra at *E*_ex_ > 781.7 eV are dominated by the scattering due to LS Co^3+^. Actually, at *E*_ex_ = 777.4 and 779.0 eV, the RIXS spectra of the HS Co^2+^ film [(a)] is much stronger than those of the LS Co^3+^ film [(c)]. We show magnified spectra in Fig. S4.

In the HS Co^2+^ film [(a)], the spectra at *E*_ex_ = 777.4, 779.0 and 779.8 eV show two intense peaks around 1.1 and 2–3 eV. The spectral feature is similar to the Co *L*_3_-edge RIXS spectra of HS Co^2+^ in CoO^18,19^. In CoO, the peak around 1.1 eV is due to excitations to the ^4^T_2g_(^4^F) states, the shoulder around 2.2 eV is due to transitions to the ^4^A_2g_(^4^F) states, and the manifold of peaks around 2.5–3.0 eV are mainly due to transitions to the ^4^T_1g_(^4^P) states^18^. The crystal field value (10*Dq*), which is the energy difference between the ^4^T_2g_(^4^F) and ^4^A_2g_(^4^F) states, is evaluated to be 0.95 eV in the HS Co^2+^ film. At 784.0 eV, broad band at ~7 eV is probably due to the charge-transfer excitations from CN^−^ to Co^2+^. In the LS Co^3+^ film [(c)], the spectra at *E*_ex_ = 781.7 eV shows intense peaks around ~2.3 eV with a shoulder structure around 3.0–6.0 eV. The spectral feature is similar to the Co *L*_3_-edge RIXS spectra of LS Co^3+^ in LaCoO_3_^20,21^, but its crystal-field excitation energy is much larger^21^. Tomiyasu *et al*. reported Co *L*_3_-edge RIXS of LaCoO_3_ single crystal with high energy resolution (~80 meV). In LaCoO_3_, the RIXS spectrum at 20 K shows intense peak around 1.3 eV with a shoulder structure around 0.6 eV. In the LS Co^3+^ film[(c)], at *E*_ex_ = 718.7 eV, the corresponding features are observed around 2.3 and 1.8 eV. Hereafter, we will refer the RIXS spectra of the HS Co^2+^ [(a)] and LS Co^3+^ [(c)] films as ϕ_2+_ and ϕ_3+_, respectively. In the mixed film [(b)], at *E*_ex_ < 779.8 eV, the spectra shows two-peak feature, which is characteristic to ϕ_2+_ [(a)]. At 784 eV, the spectrum shows a broad band around 1.0–3.0 eV and ~7 eV, whose feature is close to ϕ_3+_ [(c)].

**Figure 4:**
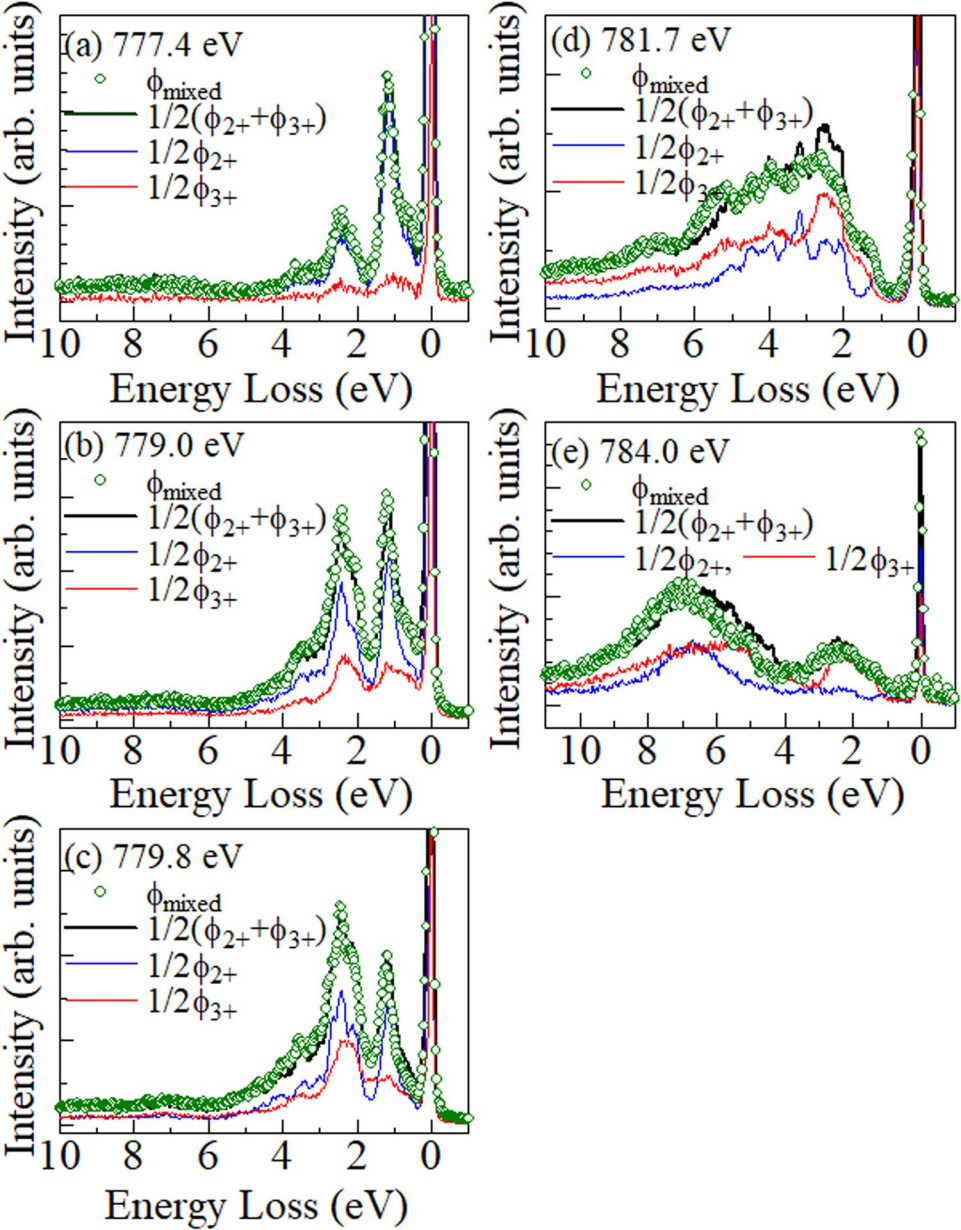
RIXS spectra (ϕ_mixed_) at *x* = 1.1 around the Co *L*_3_-edge at (**a**) 774.0 eV, (**b**) 779.0 eV, (**c**) 779.8 eV, (**d**) 781.7 eV, and (**e**) 784.0 eV. ϕ_2+_ and ϕ_3+_ are the corresponding RIXS spectra of the *x* = 1.6 and 0.0 films, respectively. ϕ_mixed_, ϕ_2+_, and ϕ_3+_ were normalized to the incident photon flux. The thick black curves represent (ϕ_2+_ + ϕ_3+_)/2.

In order to quantitatively analyze the RIXS spectra (ϕ_mixed_) of the mixed film (*x* = 1.1), we compared ϕ_mixed_ with linear combination of ϕ_2+_ and ϕ_3+_. We performed least-squares fitting of ϕ_mixed_ with a trial function: *c*ϕ_2+_ + (1 − *c*) ϕ_3+_. The adjustable parameter (*c*) were 0.48 at (a) 777.4 eV, 0.62 at (b) 779.0 eV, 0.52 at (c) 779.8 eV, 0.44 at (d) 781.7 eV, and 0.77 at (e) 784.0 eV. Except at (e) 784.0 eV, the *c* values are close to 1/2. Figure 4 shows comparison of ϕ_mixed_ with (ϕ_2+_ + ϕ_3+_)/2 at the respective *E*_ex_. The (ϕ_2+_ + ϕ_3+_)/2 spectra quantitatively reproduces ϕ_mixed_. Especially, the agreement is good at (a) 777.4 eV, (b) 779.0 eV, and (c) 779.8 eV, where the spectra are dominated by scattering due to HS Co^2+^. Utilizing the characteristics of high energy resolution, we evaluated the energy shifts (Δ*E*) of the peaks around 1.1 and 2–3 eV between ϕ_2+_ and ϕ_mixed_: Δ*E* = 0.07 and 0.00 eV at (a) 777.4 eV, 0.04 and 0.05 eV at (b) 779.0 eV, and 0.00 and 0.02 eV at (c) 779.8 eV. Thus, the local electronic state around HS Co^2+^ is invariant within the energy resolution (<100 meV) against the partial oxidization. At (d) 781.7 eV and (e) 784.0 eV, where the spectra at are dominated by scattering due to LS Co^3+^, the agreement is satisfactory except for slight difference in peak energy and intensity. At (e) 784.0 eV, the broad band around ~2.3 eV is well reproduce by ϕ_3+_. This indicates that the oxidized Co^3+^ site takes LS configuration because the ~2.3 eV peak is characteristic to the crystal-filed excitation of the LS Co^3+^ ^21^. The slight spectral difference is perhaps due to the partial oxidization of Fe^2+^ in the LS Co^3+^ (*x* = 0.0) film. In Fig. S3, we show X-ray absorption spectra around the Fe *L*_2,3_-edge. The *x* = 0.0 spectrum is significantly different from the *x* = 1.6 and 1.1 spectra, reflecting the partial oxidization of Fe^2+^.

## Discussion

Now, let us discuss the local electronic state around the Co site in the mixed (*x* = 1.1) film. The RIXS spectroscopy revealed (1) the local electronic state around HS Co^2+^ is invariant within the energy resolution (~100 meV) and (2) the oxidized Co^3+^ site takes the LS configuration in the charge process. In the discharge state, all the Co sites take the HS Co^2+^ configuration. In the oxidization process, an electron is removed from a Co site to produce an oxidized Co^3+^ state among the inherent HS Co^2+^ environment. The RIXS spectroscopy clearly indicates that the effect of the oxidized site does not reach to the neighboring Co^2+^ site. In other words, the oxidized Co^3+^ state is strongly localized. Kurihara *et al*.^22^ reported *ab initio* band calculation of Na_2_Co^2+^[Fe^2+^ (CN)_6_] and NaCo^3+^[Fe^2+^ (CN)_6_] based on the local density approximation (LDA + *U*) with the on-site Columbic repulsion. In Na_2_Co^2+^[Fe^2+^ (CN)_6_], the valence band is strongly hybridized state among the Co 3*d*, Fe3*d* and CN states. In NaCo^3+^[Fe^2+^ (CN)_6_], the valence band is characterized by purely Fe 3*d* state. The rate of orbital hybridization with the Co 3*d* and CN states is negligibly small. Similarly, the conduction band is characterized by purely Co 3*d* state. Thus, *ab initio* band calculation indicates the negligible hybridization effect of NaCo^3+^[Fe^2+^ (CN)_6_], which is consistent with the picture of localized Co^3+^ state.

Here, let us consider the coordination field effect. The lattice constant (*a*) of the fully-reduced and fully-oxidized states are 10.2 Å and 9.9 Å^8^, reflecting the difference in the ionic radius between HS Co^2+^ (*r*_HS_ = 0.745 Å) and LS Co^3+^ (*r*_LS_ = 0.545 Å). Structurally, the decrease in *a* is ascribed to the shortening of the Co − N distance. On the other hand, the RIXS spectroscopy revealed that the oxidized Co^3+^ site takes the LS configuration even in the partially-oxidized in which Co^2+^ and Co^3+^ coexist. This means that the coordination field is much stronger around Co^3+^ than that around Co^2+^. Then, the Co – N distance around Co^3+^ is considered to shorter than that around Co^2+^. These arguments reaches a picture of the partially-oxidized state, *i. e*., the electronic state is strongly localized within the range of each Co atom by making the lattice structure heterogeneous at the atomic level.

Why such a heterogeneous structure is possible in Na_*x*_Co[Fe(CN)_6_]_0.9_? We ascribed the heterogeneity to the structural flexibility in the 3D network, -Co-NC-Fe-CN-Co-. The network is fairly sparse and has 10% Fe(CN)_6_ vacancies. Actually, the density (~1.9 g/cm_3_) of Na_*x*_Co[Fe(CN)_6_]_0.9_ is much smaller as compared with that (=5.0 g/cm^3^) of layered oxides. With such a structure, the decrease in the Co − N distance around Co^3+^ in the -Co-NC-Fe-CN-Co-[]-Co-NC- ([] represents the [Fe(CN)_6_] vacancy) chain is well compensated by expansion of the vacancy space (or movement of Co along axis). In the actual 3D network, the movement of Co along chain slightly elongates the Co – Fe distance perpendicular to the chain (if the Fe site is fixed). The elongation, however, is negligibly small [=0.002 Å with the Co movement of 0.2 Å (=*r*_HS_ − *r*_LS_)]. Thus, the strong localization of the oxidized Co^3+^ state is stabilized by the [Fe(CN)_6_] vacancies. The Na_*x*_Co[Fe(CN)_6_]_0.9_ film shows good cyclability in SIB^8^. The discharge capacity is 71% of the initial value even after 100 cycles. The coulomb efficiency (>98%) remains high even after 100 cycles. The localization of the oxidized state is advantageous for the reversibility of the redox process, since the localization reduces extra reaction within the materials and resultant deterioration.

## Summary

The *L*-edge RIXS investigation of the Na_*x*_Co[Fe(CN)_6_]_0.9_ films revealed (1) the local electronic state around HS Co^2+^ is invariant within the energy resolution (~100 meV) and (2) the oxidized Co^3+^ site takes LS configuration in the charge process. We ascribed the strong localization of the oxidized Co^3+^ state to the heterogeneous lattice structure. The localization of the oxidized state is advantageous for the reversibility of the redox process, since the localization reduces extra reaction within the materials and resultant deterioration. Thus, the advanced spectroscopy with use of 3^rd^ generation synchrotron-radiation facility is a powerful tool to reveal the microscopic process within the battery materials.

## Method

### Preparation and characterization of Na_1.6_Co[Fe(CN)_6_]_0.9_ film

The electrochemical deposition of the Na_1.6_Co[Fe(CN)_6_]_0.9_ film was performed in a three-pole beaker-type cell. The working, counter, and standard electrodes were an indium tin oxide (ITO) transparent, Pt, and standard Ag/AgCl electrodes, respectively. The electrolyte was an aqueous solution containing 0.8 mmol/L K_3_[Fe(CN)_6_], 0.5 mmol/L Co(NO_3_)_2_, and 5.0 mol/L NaNO_3_. The films were deposited on the ITO electrode under potentiostatic conditions at −0:45 V vs. the Ag/AgCl electrode. The thickness of the film was around 1.4 μm, which was determined by a profilometer (Deltak-3030). The chemical composition of the film was determined by the inductively coupled plasma (ICP) method and CHN organic elementary analysis (PerkinElmer 2400 CHN Elemental Analyzer). The compound contains crystal waters as Na_1.6_Co[Fe(CN)_6_]_0.9_2.9 H_2_O. The X-ray diffraction patterns of the Na_1.6_Co[Fe(CN)_6_]_0.9_ films were obtained with a Cu Kα lines. All the reflections can be indexed with the face-centered cubic structure. The lattice constants (*a*) were 10.27 Å. The scanning electron microscopy (SEM) revealed that the films consist of crystalline grains of several hundred nm in diameter^23^.

### Battery cell

The *x* value of the Na_*x*_Co[Fe(CN)_6_]_0.9_ film was finely controlled in a beaker-type cell with two-pole configuration. The cathode, anode, and electrolyte were the film, Na metal, and M NaClO_4_ in propylene carbonate (PC), respectively. The electrochemical control was performed with a potentiostat (HokutoDENKO HJ1001SD8) in an Ar-filled glove box. The active areas of the films were about 0.5 cm^2^. The cut-off voltage was in the range of 2.0 to 4.0 V. The charge rate was about 1 C. Figure S1 shows charge curve of the Na_*x*_Co[Fe(CN)_6_]_0.9_ film. The mass of each film was evaluated from thickness, area, and ideal density. The *x* value was evaluated from the total current under the assumption that *x* = 1.6 (0.0) is in the discharge (charge) state. Thus prepared films are listed in Table 1.

### X-ray absorption spectra around the Co *L*_2,3_- and Fe *L*_2,3_-edges

The XAS measurements were conducted at TLS BL08B1 beamline at the NSRRC in Taiwan. The absorption spectra around the Co *L*_2,3_- and Fe *L*_2,3_- edges were measured in the TEY and PFY mode using an electrometer (KEITHLEY 6514) and a silicon drift detector (SDD, Amptek), respectively. The *x*-controlled Na_*x*_Co[Fe(CN)_6_]_0.9_ films were inserted into a vacuum chamber with a base pressure of 6 × 10^−8^ Torr. CoO and Fe_2_O_3_ were measured as a reference for relative energy calibration. The energy resolutions were approximately 0.3 eV. The measurement was performed at room temperature.

### RIXS around the Co *L*_3_-edge

The RIXS measurements were conducted using the AGM-AGS spectrometer at TLS BL05A1 beamline^17^ at the NSRRC in Taiwan. The *x*-controlled Na_*x*_Co[Fe(CN)_6_]_0.9_ films were inserted into a vacuum chamber with a base pressure of 2 × 10^−8^ Torr. The incident photon energy was set around the *L*_3_-edge of Co. The scattering angle defined as the angle between the incident and the scattered X-rays was 90°, and the incident angle from the surface plane was 20°. The incoming X-ray was linearly polarized with the polarization perpendicular to the scattering plane, i.e., σ polarization. The beam size of incident X-ray projected on the sample was about 0.4 mm (vertical) ×0.8 mm (horizontal). Since the injection angle is 20° off the sample surface, horizontal projection of the beam on the sample was extended to about 2 mm. The RIXS spectra were recorded with a CCD detector. To avoid the radiation damage, the incident X-ray flux was reduced by narrowing vertical slit of the monochromator and the sample position was vertically shifted by 0.2 mm at every 15 min. We confirmed that the spectral profile does not change in this radiation condition. Each spectrum was recorded for about 2 hour. The total RIXS energy resolution was ~100 meV at 780 eV. The measurement was performed at room temperature.

## Electronic supplementary material

supporting information
