# Supplementary material for: Strong localization of oxidized Co3+ state in cobalt-hexacyanoferrate
Source: Sci Rep. 2017 Nov 29;7:16579. doi: 10.1038/s41598-017-16808-1 (PMC5707369; doi:10.1038/s41598-017-16808-1)
Supplement: Supplementary file 1 — supporting information [file 41598_2017_16808_MOESM1_ESM.doc]

Supporting information

**Strong localization of oxidized Co^3+^ state in cobalt-hexacyanoferrate**

**Hideharu Niwa^1,2*^, Masamitsu Takachi^2^, Jun Okamoto^3^, Wen-Bin Wu^3^, Yen-Yi Chu^3^, Amol Singh^3^, Di-Jing Huang^3^, Yutaka Moritomo^1,2,4*^**

^1^Faculty of Pure and Applied Science, University of Tsukuba, Tsukuba 305-8571, Japan

^2^Graduate School of Pure and Applied Science, University of Tsukuba, Tsukuba 305-8571, Japan

^3^National Synchrotron Radiation Research Center, Hsinchu 30076, Taiwan

^4^Tsukuba Research Center for Energy Materials Science (TREMS), University of Tsukuba, Tsukuba 305-8571, Japan

Contact information:

Yutaka Moritomo / Hideharu Niwa

Faculty of Pure and Applied Science,

Univ. of Tsukuba, Tennodai 1-1-1, Tsukuba 305-8571, Japan

Tel +81-29-853-4337 / +81-29-853-4216

e-mail: moritomo.yutaka.gf@u.tsukuba.ac.jp / niwa.hideharu.ga@u.tsukuba.ac.jp

Fig. S1: Charge curve of the Na*_x_*Co[Fe(CN)_6_]_0.9_ film. The cut-off voltage was in the range of 2.0 to 4.0 V. The charge rate was about 1 C. The *x* value was evaluated from the total current under the assumption that *x* = 1.6 (0.0) is in the discharge (charge) state.

Fig. S2: XAS in the Co *L*_2,3_-edge region in the Na*_x_*Co[Fe(CN)_6_]_0.9_ film against Na^+^ concentration (*x*). Solid and dashed lines represents total electron yield (TEY) and partial fluorescence yield (PFY) modes, respectively. The TEY mode is surface-sensitive.

Fig. S3: XAS in the Fe *L*_2,3_-edge region in the Na*_x_*Co[Fe(CN)_6_]_0.9_ film against Na^+^ concentration (*x*). The measurements were performed in the TEY mode.

Fig. S4: RIXS spectra around the Co *L*_3_-edge of the Na*_x_*Co[Fe(CN)_6_]_0.9_ film: (a) *x* = 1.6, (b) 1.1, and (c) 0.0. The incident Photon energies are 777.4 eV (i), 779.0 eV (ii) , 779.8 eV (iii), 781.7. eV (iv) , and 784.0 eV (v). The spectra were normalized to the incident photon flux.
